# Encapsulation of the lipidated TLR7/8 agonist INI-4001 into ionic liposomes impacts H7 influenza antigen-specific immune responses

**DOI:** 10.1007/s13346-025-01917-6

**Published:** 2025-07-14

**Authors:** Fatemeh Mehradnia, Hardik Amin, Maria E. Ferrini, Haley Partlow, Timothy Borgogna, Soma Shekar Dachavaram, Kendal T. Ryter, Hélène G. Bazin, Jay T. Evans, David J. Burkhart, Blair DeBuysscher, Walid M. Abdelwahab

**Affiliations:** 1 Center for Translational Medicine-Adjuvant Research Team (CTM-ART), Missoula, MT 59812 USA; 2 Department of Biomedical and Pharmaceutical Sciences, University of Montana, 32 Campus Drive, Missoula, MT 59802 USA; 3 Department of Chemistry, University of Montana, 32 Campus Drive, Missoula, MT 59812 USA; 4 Inimmune Corporation, 1121 East Broadway, Missoula, MT 59812 USA

**Keywords:** Ionic liposomes, Vaccine adjuvant, TLR7/8 agonist, INI-4001, Influenza A virus, Hemagglutinin H7

## Abstract

Toll-like receptors (TLRs) are the best characterized family of Pattern Recognition Receptor-targeting adjuvants. Activation of TLR7/8 in particular, enhances antigen presentation by dendritic cells and macrophages, boosting Th1-mediated adaptive immune responses. However, poor pharmacokinetics and systemic toxicity of some TLR7/8 ligands hinder their clinical translation. Lipidation and incorporation of TLR7/8 ligands into liposomes can reduce systemic exposure, improve pharmacokinetics, pharmacodynamics, and presentation to immune cells. In the present study, a series of liposomal formulations incorporating a novel lipidated TLR7/8 agonist, INI-4001, were prepared to evaluate the effect of surface charge and lipid composition on the colloidal stability, cytotoxicity, innate immune activation, and adjuvant activity when combined with the recombinant Influenza A/Shanghai/1/13 (H7N9) Virus Hemagglutinin antigen (H7). The tested formulations include neutral (DOPC/Cholesterol), anionic (DOPG/Cholesterol), and cationic (DOPC/DC-Cholesterol, DOPC/GL67, DOEPC/Cholesterol, DOTAP/Cholesterol, or DOTAP + DDAB/Cholesterol) liposomes. These studies demonstrated that alongside the type and magnitude of particle surface charge, lipid composition was a determining factor in the regulation of immunogenicity and biocompatibility of INI-4001-loaded liposomes. Among the cationic liposomes evaluated, DOPC/DC-Cholesterol liposomes exhibited the lowest in vitro cytotoxicity while enhancing TNF-α induction from human peripheral blood mononuclear cells. Furthermore, murine immunization studies demonstrated that the same formulation enhanced H7-specific IgG titers and induced a Th1-polarized cell-mediated response compared to H7 antigen alone or the matched liposome lacking INI-4001. Of note, the anionic DOPG/Cholesterol INI-4001 liposomes induced a rapid response with significantly higher IgG titers after a single immunization and promoted strong Th1-polarized cellular responses, which may be advantageous in an influenza pandemic setting. These findings highlight the key role of liposome characteristics in optimizing the safety and immunogenicity of TLR7/8 agonist-adjuvanted subunit influenza vaccines.

## Introduction

In contrast to whole-cell inactivated or attenuated vaccines, subunit vaccines represent a modern approach that offers a means to develop vaccines with an excellent safety profile [1–3]. In addition, subunit vaccines can be produced with precise specifications and enhanced consistency between batches [4, 5]. Despite these advantages, subunit vaccines typically induce weaker immune responses compared to whole-cell inactivated vaccines because their highly purified antigens lack immune-stimulating components present in attenuated or inactivated vaccines [6]. Consequently, subunit vaccines require the inclusion of immune-potentiating adjuvants [7] and often necessitate multiple administrations to ensure long-lasting protection. These adjuvants boost the innate immune response and help shape the desired adaptive immune response.

Pattern recognition receptors (PRRs) are innate immune sensors expressed primarily by immune cells that detect conserved molecular motifs associated with pathogens called Pathogen-associated molecular patterns (PAMPs), such as lipopolysaccharide, unmethylated CpG DNA, or viral RNA, and initiate immune responses through downstream signaling cascades. Synthetic molecules targeting PRRs of the innate immune system are desirable adjuvant candidates. Molecules targeting PRRs induce immunity via well-defined signaling pathways, facile chemical modification for improving efficacy and safety, and potential for rapid and low-cost large-scale production with high purity [8]. Toll-like receptors (TLRs) are a major family of PRRs, which recognize and respond to PAMPs, inducing distinct immune responses by activating a wide range of downstream signaling pathways [9]. TLR7/8, which are localized in the endosome, are promising adjuvant targets as they are able to mediate the activation of the innate immune response upon recognition of exogenous single stranded RNA (ssRNA) [10]. Many synthetic TLR7/8 agonists are based on scaffolds such as 8-oxoadenine, imidazoquinoline, or purine and adenine mimetics to replicate the nucleoside-like architecture recognized by TLR7 and TLR8 [11]. Activation of TLR7/8 triggers both antiviral and proinflammatory cytokines such as IFN-α and TNF-α, respectively. In addition to increases in cytokine production, TLR7/8 activation also upregulates co-stimulatory molecules on antigen-presenting cells (APCs), resulting in the enhancement of antigen-specific T cell responses and promotion of adaptive immune responses [12].

Injection site reactogenicity as well as systemic toxicity due to the distribution of adjuvant from the injection site are the major adverse effects associated with the administration of improperly formulated water-soluble TLR7/8 agonists [13]. Therefore, strategies to retain the adjuvant at the injection site such as lipidation and particulate formulation of TLR7/8 agonists, are crucial for the development of safe and effective vaccines.

Pathogen-mimicking approaches are generally very promising for improving the efficacy of vaccine delivery systems. Pathogens including bacteria, viruses, and fungi exist in a particulate geometry in nature, thus, formulating adjuvants and subunit vaccines into particulate form can enhance their interaction and uptake by APCs, ultimately improving immune responses [14, 15]. These strategies exploit the particulate nature, multivalency, and endosomal uptake pathways that closely resemble those of actual pathogens. This improves dendritic cell (DC) targeting, promotes cross-presentation, and enhances T-cell priming. Such approaches offer several advantages, including increased stability and protection of the adjuvant/antigen from degradation, prolonged delivery and sustained exposure to immune cells by delaying clearance from the injection site, and targeted delivery to specific APC subsets, thereby closely mimicking natural infection. Furthermore, they enable controlled release and optimized intracellular trafficking of both the adjuvant and antigen [15–17]. Virus like particles, polymeric nanoparticles, liposomes, nano-emulsion are some of the examples of pathogen mimicking systems that has been studied in past [18].

Liposomes are versatile nanoparticulate-based delivery systems that have been approved for clinical applications since 1995 [19]. Liposomes are spherical vesicles composed of lipid bilayers formed through self-assembly of specific amphiphilic lipids in aqueous solutions. In our approach, liposomes of varied charge mimic key pathogen-associated features, such as nanometer-scale size, surface charge influencing uptake by APCs, and presentation of antigen and adjuvant, making them effective pathogen-mimicking carriers. Furthermore, liposomes can improve the immunogenicity of vaccines by stabilizing both antigen and adjuvant, enhancing their cellular and tissue uptake and localizing them at the site of administration leading to less systemic toxicity [20]. The versatility of liposomes is a great advantage, as it is possible to control and fine tune their structure and physicochemical characteristics through careful optimization of the lipid composition to accommodate the chemical properties of the adjuvant and antigen and the desired immune response [21, 22]. Recent studies have demonstrated the efficacy of liposomal subunit HA vaccine formulations against pandemic influenza, such as the work by Li et al. [23], which highlights the potential of liposome-based delivery systems in eliciting protective immune responses during influenza outbreaks.

**Fig. 1:**
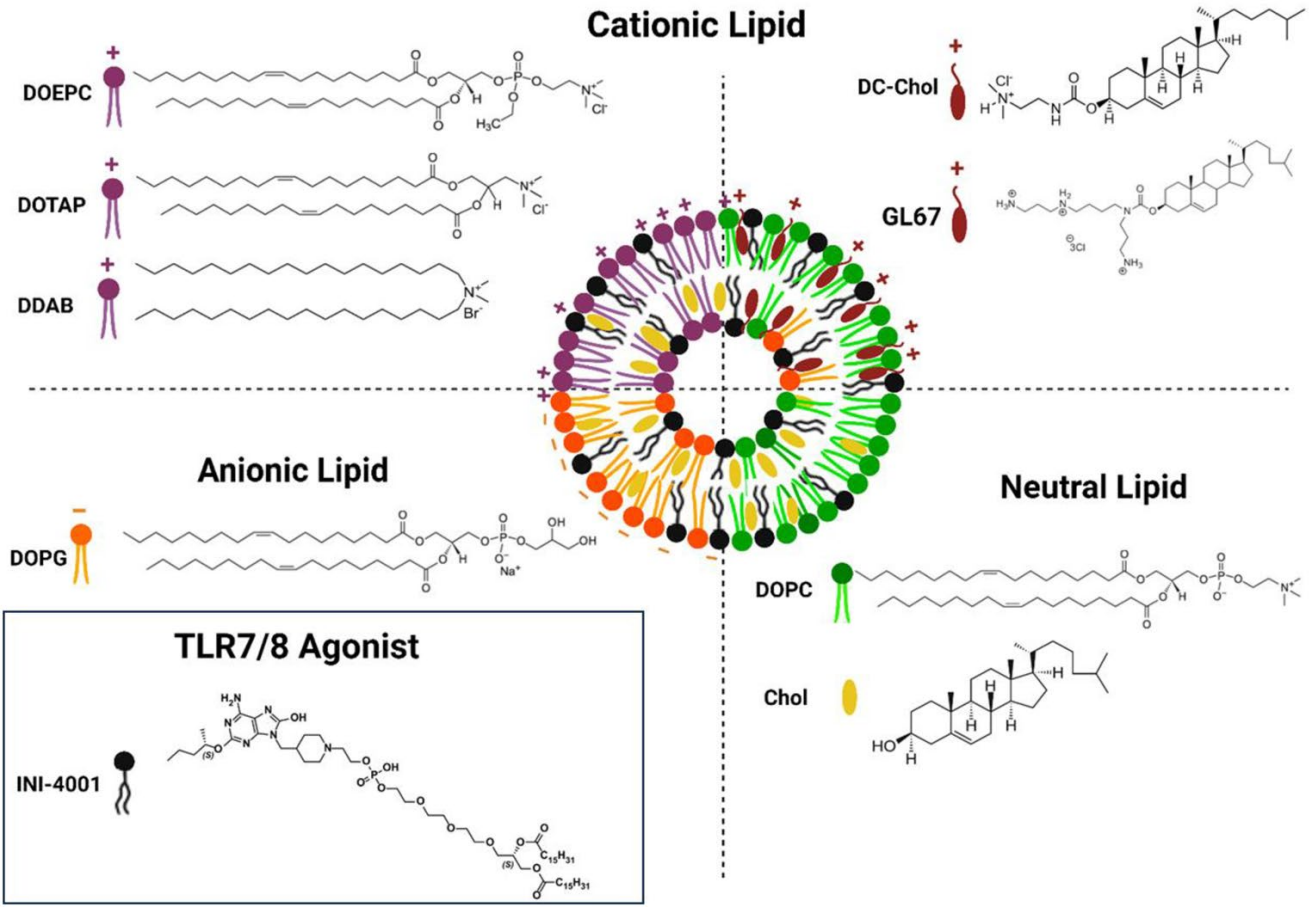
Structures of INI-4001 and lipids used to prepare the ionic liposomes

In the present study, various liposomal formulations of the novel synthetic lipidated oxoadenine TLR7/8 ligand, INI-4001 [24, 25] (Fig. 1) were prepared and thoroughly evaluated, both in vitro and in vivo. The aim of this study was to examine the effect of liposome charge (neutral, anionic, cationic) and lipid composition on the antigen-specific humoral and cellular immunogenicity of INI-4001 liposomes when combined with the recombinant Influenza A/Shanghai/1/13 (H7N9) Virus Hemagglutinin antigen (H7) [26].

## Materials and methods

### Materials

INI-4001 (phospholipidated 6-amino-2-butoxy-9-[(1-hydroxyethyl-4-piperidinyl)-methyl]-7,9-dihydro-8-H-purin-8-one) was synthesized and processed to over 99% purity as described previously [27, 28]. The H7 protein was obtained from Krammer’s lab at the Icahn School of Medicine at Mount Sinai and was produced and purified as previously described [26]. All the lipids including Cholesterol (Chol), 1,2-dioleoyl-*sn*-glycero-3-phosphocholine (DOPC), and 1,2-dioleoyl-*sn*-glycero-3-phosphoglycerol (DOPG), cholesteryl 3β-*N*-(di­methyl­amino­ethyl)­carbamate hydrochloride (DC-Chol), N4-Cholesteryl-Spermine HCl Salt (GL-67), 1,2-dioleoyl-3-trimethylammonium-propane (DOTAP), 1,2-dioleoyl-*sn*-glycero-3-ethylphosphocholine (chloride salt) (DOEPC), dimethyldioctadecylammonium (bromide salt) (DDAB) were purchased from Avanti Polar Lipids Inc. (Birmingham, AL, USA). The solvents were obtained from Fisher Scientific International L.L.C. (Massachusetts, USA). All reagents were of analytical grade and used without further purification. See Fig. 1 for structures of the TLR7/8 agonist INI-4001 and the lipids used in this study.

### Preparation of liposomes

Liposomes of various charge (neutral, anionic, and cationic) as presented in Table 1 were prepared via a thin-film hydration method [29]. Briefly, stock solutions of structural lipid, sterol lipid, and INI-4001 in chloroform were added to round bottom glass vials. The organic solvent was removed using a vacuum concentrator under reduced pressure (SpeedVac) of 100 mTorr at 30 °C for 4 h. The resulting thin films were hydrated in a buffer composed of 50 mM sodium phosphate/100 mM NaCl at pH 7.4. The targeted concentrations of INI-4001, structural lipid, and sterol lipid were 1.75 mM, 50 mM, and 25 mM, respectively. The samples were sonicated initially using a bath sonicator (Elma 933) at 37 kHz frequency, on sweeping mode, 30 °C for 30 min, followed by a focused ultrasonicator (Covaris S2) at 10% duty cycle, intensity of 8 and 500 cycles/burst with intermittent gaps of low power for rest at 20 °C until particle size below 100 nm was obtained or until particle size remained unchanged upon further sonication. The formulations were sterile filtered through 0.22 μm syringe filters (Millex GV PVDF filter) and stored at 4 °C. To avoid any unwanted TLR agonism due to the presence of endotoxin, the formulation process was performed under aseptic conditions in a BioChemGard biosafety cabinet using depyrogenated glassware and endotoxin-free consumables.

**Table 1:** Composition and charge of the INI-4001-loaded ionic liposomal formulations

Liposomal formulation	Structural lipid (Conc.)	Sterol lipid (Conc.)	Surface charge
INI-4001/**DOPG**/Chol	DOPG (50 mM)	Cholesterol (25 mM)	Anionic
INI-4001/**DOPC**/Chol	DOPC (50 mM)	Cholesterol (25 mM)	Neutral
INI-4001/DOPC/**DC-Chol**	DOPC (50 mM)	DC-cholesterol (25 mM)	Cationic
INI-4001/DOPC/**GL-67**	DOPC (50 mM)	GL-67 (25 mM)	Cationic
INI-4001/**DOEPC**/Chol	DOEPC (50 mM)	Cholesterol (25 mM)	Cationic
INI-4001/**DOTAP**/Chol	DOTAP (50 mM)	Cholesterol (25 mM)	Cationic
INI-4001/**DOTAP + DDAB**/Chol	DOTAP (45 mM) + DDAB (5 mM)	Cholesterol (25 mM)	Cationic

### Characterization of liposomes

#### Particle size and zeta potential

Z-average particle size and polydispersity of the formulations were determined via dynamic light scattering (DLS, Malvern Zetasizer Nano-ZS) by 1:10 dilution of the formulations in sterile water for irrigation (WFI). Zeta-potential was measured in folded capillary cells on the Malvern Zetasizer by diluting samples 1:20 in WFI. All measurements were calculated as the mean ± SD (*n* = 3 independent replicates).

#### Particle morphology

A Talos Arctica cryogenic electron microscope equipped with a FEI Ceta 16 M CMOS camera was used as a courtesy of the Multiscale Microscopy Core (MMC), Oregon Health & Science University (OHSU) with technical support from the OHSU-FEI Living Lab and the OHSU Center for Spatial Systems Biomedicine (OCSSB). Before imaging, samples were transferred to Quantifoil EM grids and frozen in liquid ethane using a Vitrobot. ImageJ software was used to process the obtained images and determine the particles sizes.

#### Adjuvant concentration

The concentration of INI-4001 in each formulation was determined by RP-HPLC using a Waters 2695 separations module and a 2489 UV/Vis detector. A 9:1 *v/v* THF: methanol solution was used to dissolve the liposomal formulations. The dissolved samples were then eluted on an ACE 3 C8 50.0 × 3.0 mm id column with a Phenomenex C8 guard column. The buffer stock solution was composed of 250 mM TBAOH (tetrabutylammonium hydroxide) in HPLC water adjusted to pH 6.0 using concentrated phosphoric acid. The composition of Mobile phases A and B was as follows: 2% v/v TBAOH stock and 8% v/v ACN in HPLC water as mobile phase A, and 2% v/v TBAOH in ACN as the Mobile phase B. The reverse phase gradient run for each sample injection at 0.8 mL/min, given in T (min), was as follows: T0%A 95%B 5, T10%B 100, T15%B 100, T17.1%A 95%B 5, T20%A 95%B 5. The absorbance of INI-4001 was measured at 254 nm. The concentration of INI-4001 was determined by peak area based on interpolation from a seven-point dilution series of the corresponding standard. This method was developed based on a previously validated protocol and showed linearity and precision within the tested range of INI-4001 concentration.

#### Stability studies

To determine the effect of storage time and temperature on the colloidal stability of the developed liposomes, formulations were stored at 4, 25 and 40 °C for 3 months and the changes in the average size and zeta potential of the particles were recorded using a Malvern Zetasizer as described in the “Particle size and zeta potential” section. All measurements were calculated as the mean ± SD (*n* = 3 independent replicates).

### In vitro cytotoxicity and innate cytokine production from hPBMCs

Cytokine production from human peripheral blood mononuclear cells (hPBMCs) post incubation with different formulations was measured using commercially available ELISA kits. Briefly, blood was collected from healthy adult volunteers with informed consent and University of Montana IRB approval in accordance with HHS guidelines, IRB number 43-16. Histopaque 1,077 (Sigma) gradient separation method was used to isolate hPBMCs from whole blood. The isolated hPBMCs were then cultured in RPMI 1640 culture media (Invitrogen, Grand Island, NY) supplemented with 10% FBS (Sigma) and antibiotics (Pen/Step/Glut, Invitrogen) at 0.5 × 10^6^ cells/well density in 96-well plate and stimulated with the formulations of INI-4001 from 0.1 to 100 µM. Supernatants were collected 24 h after treatment and cytokine production was measured by human IFN-α VeriKine ELISA kit (Pestka Biomedical Laboratories, Inc., Piscataway, NJ) and human TNF-α DuoSet (R&D Systems, Minneapolis, MN, USA) ELISA kit. Cells from the same wells were assayed for cellular viability using a CellTiter-Glo (Promega Corp., Madison, WI, USA) cell viability assay according to manufacturer’s recommendations.

### In vivo vaccine studies

Groups of 7 to 8 BALB/c (6–8-week-old) mice from Jackson Laboratory were used in this study. Animals were handled in accordance with NIH Office of Lab Animal Welfare guidelines under Institutional Animal Care and Use Committee (IACUC) approved protocols (AUP 015–19). Mice were immunized three times 14 days apart by intramuscular injection (IM) in the hind limb with 1 µg of H7 and 10 µg of liposomal INI-4001 formulations. Blood was collected at 14 days post-primary and post-secondary injection and centrifuged at 10,000× g in Microtainer Serum Separator Tubes (BD Biosciences) to obtain serum, which was stored at − 20 °C. At five days post-tertiary injection, the mice were euthanized and the draining lymph nodes (dLNs) (inguinal and popliteal LNs on the injection side) and spleens were collected.

#### Serum antibody titers determination

Serum levels of H7-specific IgG antibody titers were measured as follows. ELISA plates were prepared by coating with 100 µL of H7 at 2 µg/mL, washing in 0.05% Tween-20 in PBS, and blocking with SuperBlock (Scytek Laboratories). Plates were then incubated with serum (serially diluted) for 2 h followed by an incubation with anti-mouse IgG-HRP secondary antibody (Southern Biotech, Birmingham Al, USA). The antigen specific antibodies were detected by incubating with TMB substrate (BD Biosciences) for 20 min then stopped with H_2_SO_4_. Absorbance was measured at 450 nm using a Molecular Devices SpectraMax 190 microplate reader, and antibody titers were determined by calculating the area under the curve (AUC).

#### T-cell restimulation cytokine analysis

Lymph nodes were mechanically dissociated with sandblasted slides and filtered as necessary prior to plating. For spleen samples, red blood cells were lysed by incubation with red blood cell lysis buffer (Sigma-Aldrich, St. Louis, MO, USA) for 5 min followed by washing in HBSS. 2 x 10^6^ cells per well were plated in complete RPMI 1640 in a 96-well plate, and incubated with 10 µg/mL H7 antigen for 72 h. Supernatants were harvested for measurement of cytokine secretion by MesoScale Discovery (MSD) U-PLEX Assay Platform, assaying for IFN-γ, IL-17, TNF-α, and IL-5.

### Statistical analysis

Statistical analysis was performed using GraphPad Prism 10 software. Statistical significance was determined by ordinary one-way ANOVA with Brown-Forsythe and Barlett’s tests for residuals. Post-hoc comparison of means (*p* < 0.05 *, < 0.01 **, < 0.001 ***, and <0.0001 ****) was performed using Dunnett’s test.

## Results and discussion

The lipidated structure of INI-4001 (calculated logP of 8.5) makes it suitable for incorporation into various lipid-based formulations. In this work, various ionic liposomal formulations of the TLR7/8 agonist, INI-4001, of different composition and charge characteristics (Table 1) were prepared via a thin film hydration/sonication technique to examine the effect of these variables on the immunogenicity of INI-4001 adjuvanted H7 vaccines (Fig. 1, schematic representation of INI-4001 ionic liposomes). These liposomes were formulated at a relatively high INI-4001 concentration of 2 mg/ml to determine any limitation with adjuvant incorporation into these different liposome compositions. The developed liposomes were characterized for adjuvant loading, physicochemical properties and colloidal stability, and evaluated both in vitro and in vivo for safety and immunogenicity.

### Characterization of liposomes

**Fig. 2:**
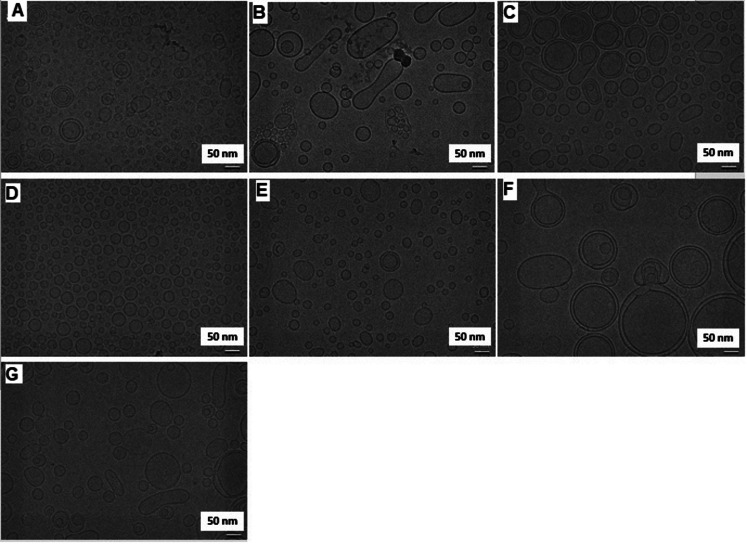
Cryo-TEM images of various INI-4001-loaded liposomal formulation with structural lipid: sterol lipid molar ratio of 2:1; **A**) DOPG/Chol, **B**) DOPC/Chol, **C**) DOPC/DC-Chol, **D**) DOPC/GL67, **E**) DOEPC/Chol, **F**) DOTAP/Chol, and **G**) DOTAP + DDAB/Chol. The data was obtained from samples analyzed approximately four weeks after preparation and filtration through a 0.22 μm sterile filter

The physicochemical properties of all liposomal formulations were assessed by measuring particle size, polydispersity index (PDI), and zeta potential using DLS. The characterization results are summarized Table 2. Ionic liposomes with or without INI-4001 of various compositions were successfully prepared into uniform particles of less than 80 nm in diameter with narrow size distribution (PDI ranged from 0.118 to 0.274) as determined by DLS measurements (Table 2). Additionally, all ionic liposomes demonstrated the expected charge as depicted in Table 1 based on the corresponding composition where the loading of anionic INI-4001 resulted in a slight decrease in surface charge of the cationic liposomes (Table 2). The particle size, shape and lamellarity of these liposomes were characterized using cryo-TEM imaging (Fig. 2). The INI-4001-loaded formulations primarily formed unilamellar liposomes with a distinct bilayer except for DOTAP/Chol formulation which predominately formed multilamellar liposomes likely due to the distinct larger packing parameters of the DOTAP lipid [30] (Fig. 2f). No particulate aggregates were observed in any of the formulations which, along with the formation of liposomes with distinct bilayer, suggests that INI-4001 adjuvant is incorporated in the liposome bilayer. Similar to the DLS measurements, all formulations predominately formed particles smaller than 100 nm in diameter except for the DOTAP/Chol formulation (Fig. 2f) in which particles larger than 100 nm were predominant by the time of imaging (approximately four weeks after preparation and filtration through a 0.22 μm sterile filter). However, the inclusion of DDAB at a 1:9 molar ratio to DOTAP in this formulation resulted in the formation of smaller and more stable unilamelar liposomes (Fig. 2g). DOPC/Chol formulation (Fig. 2b) had a noticeable population of elongated vesicles. DOPC/DC-Chol (Fig. 2c) and DOTAP + DDAB/Chol (Fig. 2g) liposomes also showed the presence of similar elongated vesicles, albeit to a lesser extent. The appearance of elongated vesicles in these samples may be attributed to mechanical forces exerted during sterile filtration through a syringe filter, or it could be a result of colloidal instability that developed over the four weeks prior to when these images were taken.

**Table 2:** Summary of characterization results of the ionic liposomal formulations with or without INI-4001. Data were taken on day 1 after preparation and sterile filtration and presented as the mean ± sd (*n* = 3 independent replicates)

Formulation	Z-Average diameter (nm)	PDI	Zeta Potential (mV)	INI-4001 conc. (µg/ml)	INI-4001 recovery (%)
DOPG/Chol	56.4 ± 0.8	0.241	-36.5 ± 1.7	-	-
INI-4001/DOPG/Chol	59.7 ± 0.6	0.201	-46.1 ± 2.4	1627 ± 69	81.3 ± 3.4
DOPC/Chol	74.2 ± 1.4	0.213	-1.9 ± 0.8	-	-
INI-4001/DOPC/Chol	71.8 ± 0.6	0.238	-2.6 ± 0.5	933 ± 235	46.6 ± 11.7
DOPC/DC-Chol	63.8 ± 1.2	0.274	21.9 ± 2.8	-	-
INI-4001/DOPC/DC-Chol	61.8 ± 0.8	0.214	18.7 ± 1.2	1329 ± 945	66.4 ± 4.7
DOPC/GL-67	51.0 ± 0.4	0.118	24.5 ± 0.43	-	-
INI-4001/DOPC/GL-67	63.4 ± 0.8	0.236	24.6 ± 1.6	1369 ± 88	68.4 ± 4.3
DOEPC/Chol	40.1 ± 0.9	0.214	34.2 ± 2.1	-	-
INI-4001/DOEPC/Chol	57.6 ± 0.6	0.227	32.2 ± 3.5	1712 ± 137	85.6 ± 6.8
DOTAP/Chol	55.11 ± 0.6	0.142	38.9 ± 1.3	-	-
INI-4001/DOTAP/Chol	71.0 ± 0.2	0.193	36.0 ± 0.94	904 ± 76	45.2 ± 3.8
DOTAP+DODAB/Chol	63.1 ± 0.6	0.178	37.4 ± 1.5	-	-
INI-4001/DOTAP + DODAB/Chol	78.2 ± 2.0	0.190	34.9 ± 2.6	710 ± 107	35.5 ± 5.3

### Recovery of INI-4001 after sterile filtration

The palmitoyl lipidated nature of INI-4001 allows it to be stably incorporated within the liposomal bilayer. However, the charge and lipid composition of the liposome may also affect its encapsulation efficiency. To evaluate the effect of these parameters on adjuvant recovery, INI-4001 concentration in each formulation was measured via RP-HPLC post-filtration through a 0.22 μm pore size syringe filter (Table 2). The results demonstrated that liposome charge was not a determining factor in INI-4001 recovery where both cationic DOEPC/Chol and anionic DOPG/Chol liposomes showed the highest INI-4001 recovery of 85.6% and 81.3%, respectively. In contrast, formulations with larger particle sizes such as DOPC/Chol, DOTAP/Chol, and DOTAP + DDAB/Chol had the lowest adjuvant recovery of 46.6%, 45.2% and 35.3%, respectively. This could be due to the tendency of these formulations to aggregate or form larger particles during the preparation process, as well as after sterile filtration and storage, as confirmed by cryo-TEM imaging (Figs. 2b, g, f).

The ability of INI-4001 to incorporate into various liposomes with different charges and compositions highlights its versatility for use in lipid-based nanoparticles.

### Assessment of colloidal stability of the lead INI-4001 liposomes

**Fig. 3:**
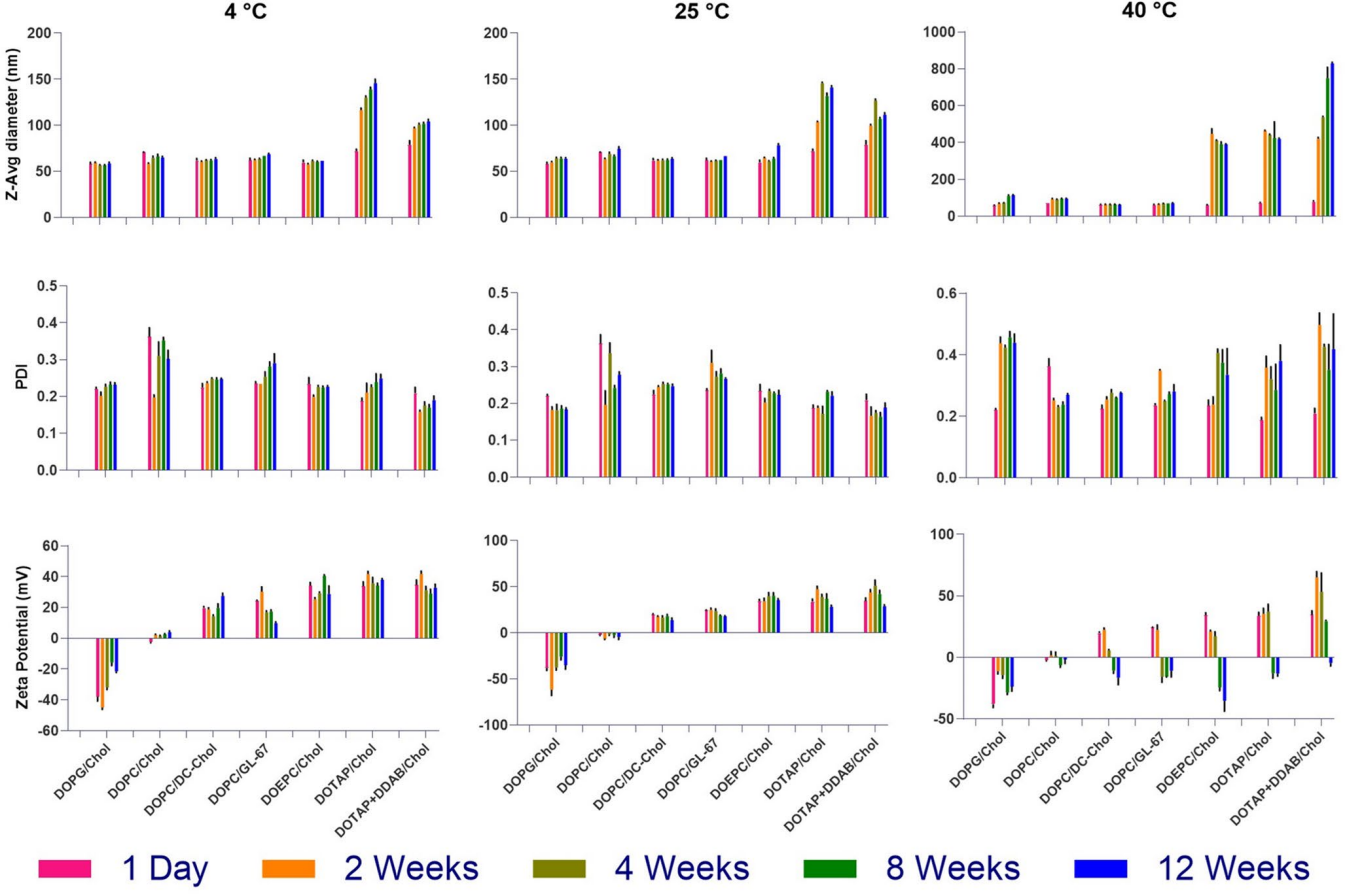
Monitoring of z-average particle size, PDI, and zeta potential of INI-4001-loaded ionic liposomes over 12 weeks of storage at 4, 25 and 40 °C. Stability data are presented as the mean ± SD (*n* = 3 independent replicates)

The effect of storage time and temperature on the colloidal stability of the liposomes was determined via monitoring the changes in particle size and zeta potential over three months of storage at 4, 25 and 40 °C. Except for DOTAP-based liposomes, all formulations demonstrated great stability for at least three months when stored at 4 and 25 °C with little or no change in particle size and zeta potential (Fig. 3). The addition of DDAB lipid to DOTAP/Chol liposomes considerably improved its stability. The average particle size and PDI of formulations containing cationic sterol lipids including DC-Chol and GL-67 remained stable at 40 °C despite a decrease in zeta potential. In contrast, liposomes containing cationic structural lipid (DOEPC, DOTAP, DDAB) lost their structural integrity and demonstrated larger particle sizes after two weeks of storage at 40 °C. This elevated temperature was used as a stress condition to assess the robustness of the formulations beyond standard storage conditions. The reduced stability of liposomes containing cationic structural lipids such as DOEPC, DOTAP, and DDAB is likely due to the labile nature and higher packing parameters of these lipids, which may promote vesicle fusion, formation of non-lamellar structures, or aggregation under thermal stress [31–33]. Despite demonstrating excellent particle size and PDI stability, INI-4001-loaded anionic DOPG/Chol liposomes exhibited a consistent decrease in zeta potential over time, even when stored at 4 °C, dropping from approximately − 46 mV on day one to around − 21 mV after four weeks of storage (Fig. 3). This may be due to the dynamic rearrangement of lipid/adjuvant components, changing the exposure of the ionic groups and their density at the lipid bilayer surface. Although we did not evaluate the chemical stability of INI-4001 when formulated into liposomes in this study, the observed colloidal stability over three months at 4 °C and 25 °C suggests the structural integrity of the liposomal formulations was maintained. Future studies are warranted to assess long-term chemical stability of INI-4001 under accelerated and real-time conditions.

Based on these results, the DOTAP/Chol formulation was eliminated from subsequent experiments due to its poor colloidal stability, even when stored at 4 °C (Fig. 3).

### In vitro cytotoxicity and innate cytokine production from hPBMCs

**Fig. 4:**
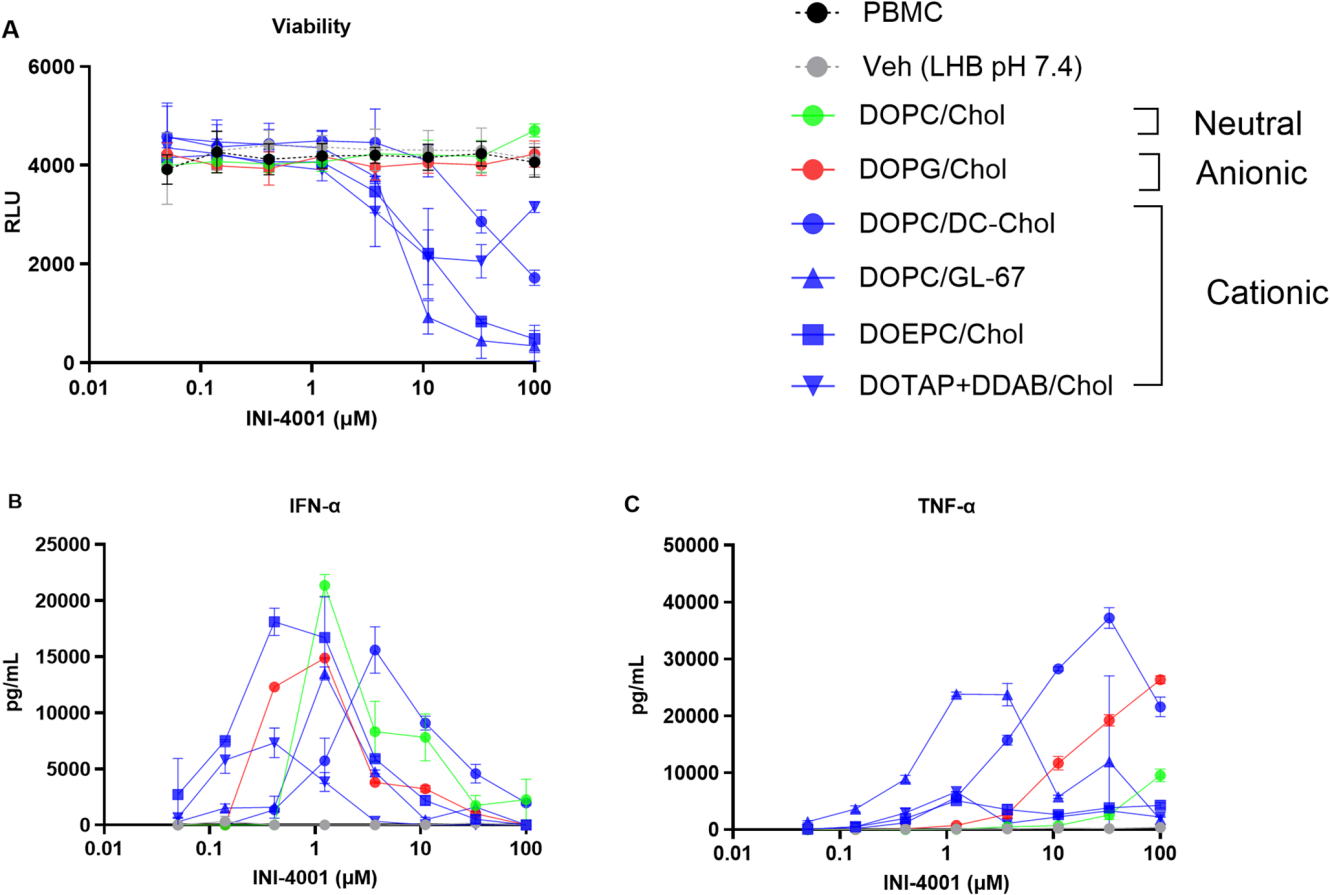
INI-4001 ionic liposomes impact cell viability (A) as well as IFN-α (B) and TNF-α (C) induction in hPBMCs. Cell viability was assessed using Cell Titer Glo. Freshly isolated hPBMCs were stimulated for 24 hours with vehicle (liposome hydration buffer (LHB), pH 7.4) or ionic liposomes containing INI-4001. IFN-α and TNF-α were quantified by ELISA from hPBMC supernatants. Data displayed is representative of three biological replicates as the mean ± SD (n = 3).

Particle charge and lipid structure can influence the uptake of liposomes by immune cells, thereby affecting trafficking, activity and cytotoxicity [34]. Cellular uptake of cationic liposomes occurs through electrostatic interaction between the anionic surface of the cell membrane and the cationic lipid, whereas anionic liposomes are taken up by macrophages through scavenger receptors [35]. Furthermore, Takano et al. [36] showed there was a significant correlation between cationic liposomal association with cells and degree of apoptosis. Studies have also indicated that reactive oxygen species (ROS) production and intracellular thiol levels are key factors in induction and regulation of apoptosis by cationic liposomes via a mitochondrial pathway [37, 38]. The cytotoxicity and adjuvant activity of the INI-4001 liposomal formulations were determined in vitro via treatment of hPBMCs with serial dilutions of INI-4001-loaded liposomes for 1 day followed by evaluation of IFN-α (a primary readout for TLR7 activation) and TNF-α (a primary readout for TLR8 activation) by ELISA and viability was determined by Cell Titer Glo assay (Fig. 4).

The neutral (DOPC/Chol) and anionic (DOPG/Chol) liposomes demonstrated the highest cell viability relative to the cationic liposomes (Fig. 4a). The viability of the cells treated with up to 100 µM of these INI-4001 liposomal formulations was comparable to the untreated control and vehicle treated cells. Among the cationic liposomes, DOPC/DC-Chol with moderate cationic charge (< 20 mV) induced no toxicity up to 11 µM adjuvant concentration. The other cationic liposomes showed no toxicity up to 3.5 µM of adjuvant but their toxicity increased considerably at higher concentrations. These findings suggest that the composition of the cationic liposomes may play a key role in determining the liposome’s effect on cell viability.

IFN-α is a cytokine capable of enhancing antiviral immunity and induction of Th1 polarized immune responses. TLR7 mediated induction of an IFN-α response can upregulate the expression of interferon regulatory factors transcription factors which mediate innate and adaptive immunity [39]. IFN-α production appears to be influenced by liposome surface charge and lipid composition. INI-4001 neutral (DOPC/Chol) and cationic sterol lipid-based (DOPC/GL-67 and DOPC/DC-Chol) liposomes demonstrated lower potency in IFN-α production compared to the anionic (DOPG/Chol) and cationic structural lipid-based (DOEPC/Chol and DOTAP + DDAB/Chol) liposomes (Fig. 4b). TNF-α is involved in controlling both innate and adaptive immune responses to viral infections. As an early cytokine, TNF-α is generated in response to viral infections through pathogen recognition receptors like TLR8. hPBMCs treated with the cationic sterol lipid-based (DOPC/GL-67 and DOPC/DC-Chol) liposomes showed the highest level of TNF-α production among all the formulations tested (Fig. 4c). The other cationic liposomes (DOEPC/Chol and DOTAP + DDAB/Chol) with higher surface charge exhibited the lowest levels of TNF-α release. Interestingly, at concentrations exceeding 10 µM, DOPG/Chol liposomes with a negative surface charge induced robust TNF-α production, though not to the same extent as the sterol lipid-based cationic liposomes (Fig. 4c). This observation suggests that cellular uptake of the formulations and TNF-α production was more influenced by the composition of ionic lipid rather than its charge. Although it is expected that cationic liposomes have a better cellular uptake due electrostatic interaction between the anionic surface of the cell and the cationic surface charge of liposome, anionic liposomes have also shown a strong uptake by macrophages through scavenger receptors as mentioned earlier [40].

An inverse correlation was observed between IFN-α and TNF-α induction among the tested cationic liposomes. DOEPC/Chol and DOTAP + DDAB/Chol liposomes were strong inducers of IFN-α but elicited minimal TNF-α. In contrast, DOPC/DC-Chol and DOPC/GL-67 liposomes induced higher levels of TNF-α than IFN-α. These differences between IFN-α and TNF-α activation may arise from the liposomes being taken up by various types of APCs found within the hPBMC populations, which can result in unequal stimulation of TLR7 versus TLR8. TLR7 is predominantly expressed in plasmacytoid DCs (pDCs) and regulate the expression of IFN-α whereas TLR8 is primarily expressed in macrophages, monocytes, mast cells, mDCs, and neutrophils in humans [41]. Our observation confirmed that the capability of INI-4001 as a TLR7/8 agonist in inducing immunogenicity, via IFN-α and TNF-α production, can be influenced by the physicochemical properties of the formulation especially the lipid structure and composition. These properties can determine the fate of liposome in terms of targeting specific APCs as well as dictating the rate and mechanism of cellular uptake (membrane fusion or endocytosis).

### In vivo immunogenicity evaluation

It was hypothesized that the incorporation of INI-4001 in liposomes of various charge and lipid composition would impact the immunogenicity and safety of INI-4001 adjuvanted H7 vaccines and could further improve the antigen-specific humoral and cellular immune responses. Previous studies have shown that cationic liposomes, in particular, can enhance the potency of TLR7/8 agonists [42–44]. To investigate this hypothesis, a series of four cationic (DOPC/DC-Chol, DOPC/GL67, DOEPC/Chol, DOTAP + DDAB/Chol), one anionic (DOPG/Chol), and one neutral (DOPC/Chol) INI-4001 liposomes and their non-adjuvanted counterparts were combined with the H7 influenza antigen and administered intramuscularly to mice in a series of three injections 14 days apart. Vaccination was conducted using a 1 µg dose of H7 and 10 µg dose of INI-4001. Blood was collected 14 days post-primary (14dp1) and 14 days post-secondary (14dp2) injections to determine H7-specific antibody responses. Five days post-tertiary injection, lymph nodes were harvested to characterize H7-specific T cell responses induced by each formulation.

**Fig. 5:**
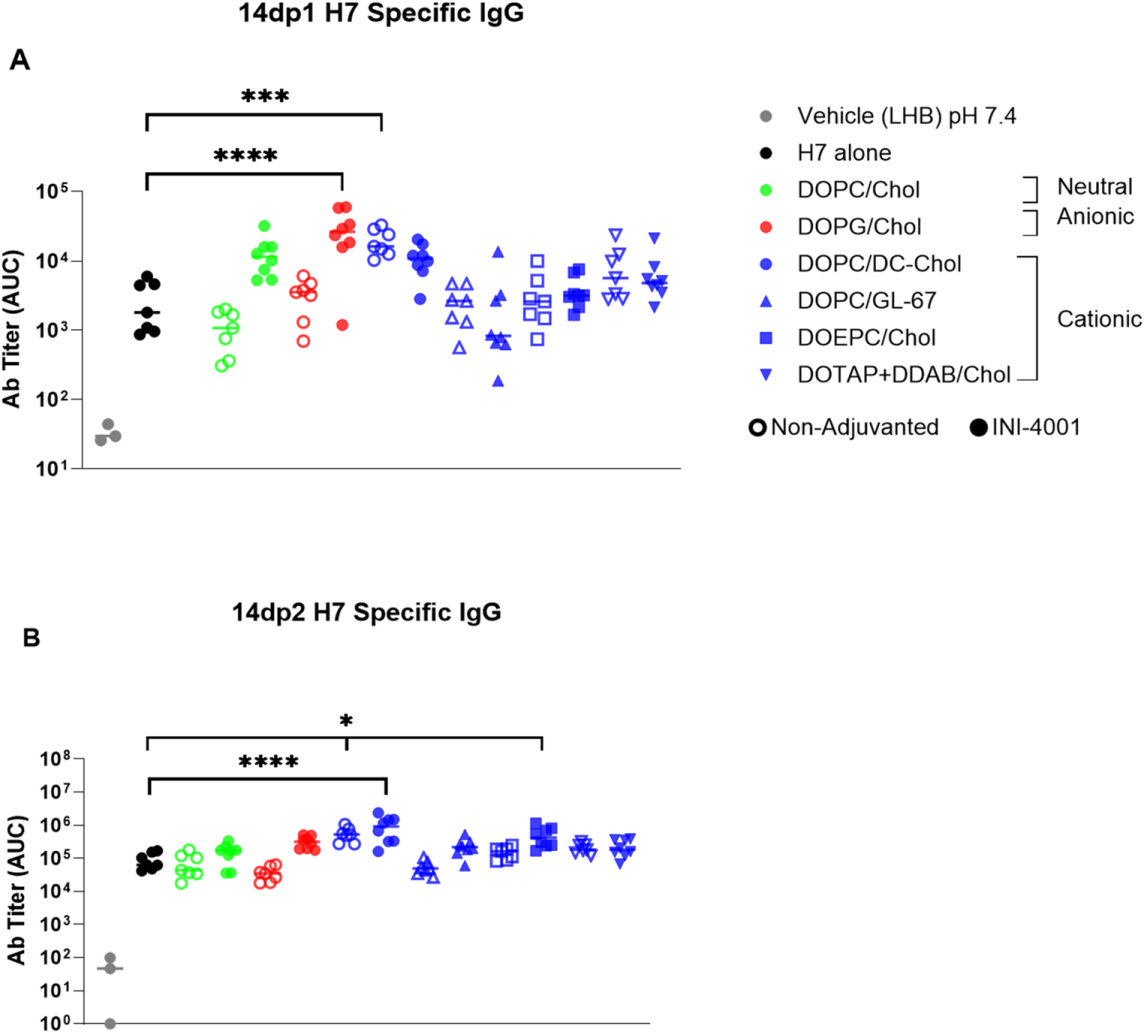
Immunogenicity evaluation of ionic liposomes with INI-4001 (solid shapes) or without INI-4001 (open shapes) 14-day post vaccination. Serum IgG antibody titers determined by ELISA (*n* = 7–8). (**A**) 14-day post-primary titers, (**B**) 14-day post-secondary titers. One-way ANOVA with post hoc Dunnett test; comparisons shown are between H7 and the different formulations. * *p* < 0.05; *** *p* < 0.001; **** *p* < 0.0001

As shown in Fig. 5a, the anionic INI-4001 DOPG/Chol liposomes induced the highest H7-specific IgG serum titers 14dp1, which was significantly improved compared to antigen alone and DOPG/Chol liposomes. This suggests that anionic liposomes can lead to a more rapid increase in humoral immunity following a single vaccination, which may be essential to immunization in an influenza pandemic setting. This response could also be particularly valuable for vaccines targeting other RNA viruses that require rapid protection, including emerging or re-emerging pathogens such as SARS-CoV-2, RSV, or Zika virus. Anionic liposomes are preferentially taken up by macrophages through scavenger receptors [35], and some studies have shown that the uptake of anionic liposomes by macrophages was higher than that of cationic liposomes [45]. However, the robust induction of both IFN-α and TNF-α from hPBMCs (Fig. 5) suggests that DOPG/Chol liposomes were also efficiently taken up by other immune cells, including pDCs, mDCs, and monocytes. The strong induction of H7-specific IgG serum titers by anionic INI-4001 DOPG/Chol liposomes may be attributed to this enhanced uptake, leading to a more robust immune response. As expected, IgG titers increased 14dp2 immunization (Fig. 5b) compared to 14dp1 immunization. Except for DOTAP + DDAB/Chol, loading of INI-4001 into the tested ionic liposomes resulted in higher IgG titers 14dp2 relative to the corresponding control liposomes. Among all the evaluated formulations, the cationic INI-4001 DOPC/DC-Chol and to a lower extent DOPC/DC-Chol (without INI-4001) and INI-4001 DOEPC/Chol liposomes demonstrated significantly higher induction of H7-specific IgG serum titers relative to the H7 alone group. These results indicate that the extent of TLR7/8 agonist-induced humoral immune response can be altered by changing the liposome composition.

ELISA-based binding antibody titers provide valuable insights into the immunogenicity of the evaluated liposomal vaccine formulations, especially when used to compare relative responses across groups. However, binding does not correlate with antibody effector function. In future studies, we plan to perform Hemagglutination inhibition assays (HAI) and other functional assays to fully characterize the protective potential of the immune responses.

**Fig. 6:**
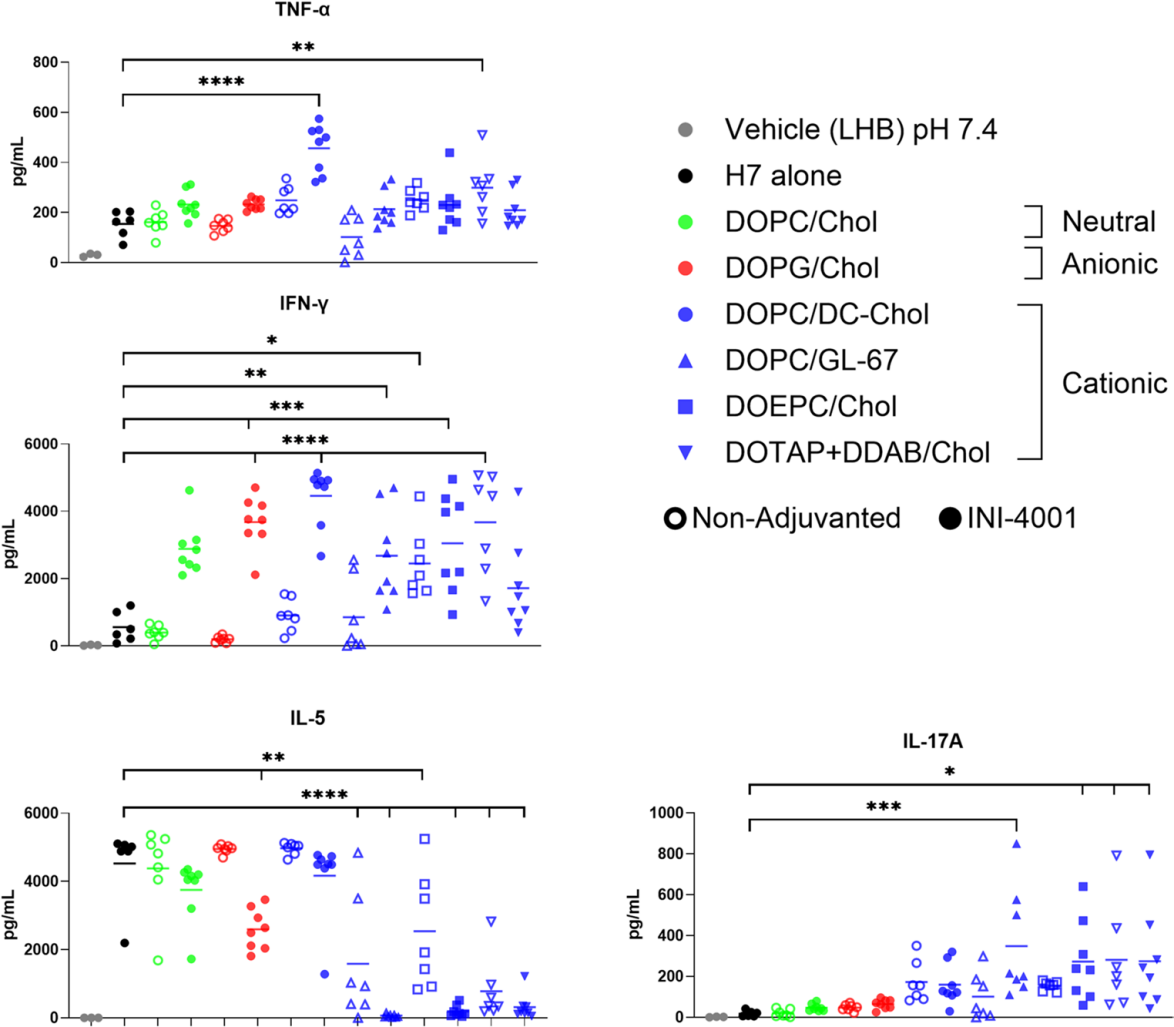
Five-day post-tertiary vaccine T-cell antigen stimulated cytokine analysis of INI-4001 ionic liposomes vaccinated mice. Cytokines were quantified from the dLNs of vaccinated mice using MSD, *n* = 7–8 mice per group. One-way ANOVA with post hoc Dunnett test; comparisons shown are between H7 and the different formulations. * *p* < 0.05; ** *p* < 0.01; *** *p* < 0.001; **** *p* < 0.0001

In addition to humoral immunity, the cellular immune response to these liposomal formulations was also investigated by measuring the production of IFN-γ, TNF-α, IL-5 and IL-17 A in response to antigen-restimulation of cells from the LNs of vaccinated mice. Considering the different impacts of formulations on the antigen-specific IgG, post-harvest antigen re-stimulation of LNs was performed to further investigate the Th1/Th2/Th17 cytokines induction by these various liposomes. Figure 6 represents the antigen-specific T cell responses in LNs harvested 5 days post-tertiary vaccination and re-stimulated ex vivo with H7 influenza antigen. Secreted cytokines were detected by MesoScale Discovery (MSD) multiplex cytokine array. The H7 antigen alone vaccinated mice were used as a control to determine the effect of adjuvant on the immune response more accurately.

With the exception of the INI-4001 DOTAP + DDAB/Chol liposomes, all the other INI-4001 liposomal formulations exhibited significantly higher H7-specific IFN-γ induction compared to the H7 alone or the non-adjuvanted liposome vaccinated mice (Fig. 6), suggestive of a Th1-biased response. TNF-α is produced by many different CD4 T-helper cell types, often described as multifunctional antigen specific T-cells. It has a role as an early inducer of inflammation to direct anti-viral responses through multiple pathways [46]. During adaptive immune responses, T cells produce TNF-α to aid in clearing the virus and restoring homeostasis in infected tissues [47, 48]. TNF-α production was also significantly increased in INI-4001 DOPC/DC-Chol liposomes vaccinated mice. Additionally, all the INI-4001-loaded liposomes resulted in less Th2-associated IL-5 production compared to the H7 alone or the non-adjuvanted liposomes. Among the evaluated liposomes, INI-4001-loaded DOPC/DC-Chol with a moderate cationic charge induced the highest production of both IFN-γ and TNF-α that was significant over the antigen alone group, indicative of a Th1-biased immunity without inducing Th-17-associated IL-17A production (Fig. 6).

Taken together, the early innate cytokine profiles, such as IFN-α and TNF-α induced in hPBMCs, seem to correlate with the magnitude and quality of the adaptive immune responses observed in vivo, including antibody titers and T cell cytokine production. Additionally, these results indicate that surface charge and lipid composition of liposomes can play a determining role in the immunomodulatory effect of TLR7/8 agonists. The DOPC/DC-Chol liposomes with moderate cationic surface charge increased both humoral and cellular immunity without significant cytotoxicity in contrast to the other liposomal formulations with higher cationic surface charges.

## Conclusion

To improve the safety and efficacy of subunit vaccines, modern vaccines incorporate PRR adjuvants alongside nanoparticle delivery systems to enhance immune cross-protection, improve humoral and cell-mediated immunity, and promote antigen dose sparing. The present report demonstrates that surface charge and composition of liposomal formulations of the TLR7/8 agonist INI-4001 are crucial in shaping their biocompatibility as well as humoral and cell-mediated immunogenicity when combined with the influenza antigen H7. For instance, the INI-4001 cationic DOPC/DC-Chol liposome with a moderate positive charge density, (zeta potential ∼20 mV) elicited enhanced TNF-α induction (associated with TLR8 activation) in vitro in hPBMCs compared to the other tested liposomes while showing low cytotoxicity. Furthermore, the same formulation significantly enhanced H7-specific IgG and Th1 cytokine production (TNF-α, IFN-γ, but not IL-5 or IL-17) compared to antigen alone and non-adjuvanted liposome controls. In contrast, liposomal formulations with high cationic charge, such as DOEPC/Chol and DOTAP + DDAB/Chol failed to promote the same level of Th1 polarized humoral and cell-mediated responses while exhibiting toxicity in hPBMCs. Notably, anionic DOPG/Chol liposomes induced rapid humoral and strong Th1-biased cellular responses but did not provide as strong of anti-H7 IgG titers 14dp2 as DOPC/DC-Chol. Taken together, cationic liposomal formulations with a moderate surface charge, such as DOPC/DC-Chol or anionic charge such as DOPG/Chol, could serve as promising delivery platforms for developing TLR7/8 adjuvanted vaccines with Th1-biased cellular and humoral immunogenicity. Such a vaccine platform has potential application in developing Influenza vaccines with enhanced efficacy and safety.

## Data Availability

The datasets generated during and/or analyzed during the current study are available from the corresponding author on reasonable request.

## Abbreviations

ACN: Acetonitrile
Chol: Cholesterol
DCs: Dendritic cells
DMSO: Dimethyl sulfoxide
DOPC: 1,2-dioleoyl-sn-glycero-3-phosphocholine
DOPG: 1,2-dioleoyl-sn-glycero-3-phosphoglycerol
DC-Chol: Cholesteryl 3β-N-(di­methyl­amino­ethyl)­carbamate hydrochloride
GL-67: N4-cholesteryl-spermine HCl Salt
DOTAP: 1,2-dioleoyl-3-trimethylammonium-propane
DOEPC: 1,2-dioleoyl-sn-glycero-3-ethylphosphocholine (chloride salt)
DDAB: Dimethyldioctadecylammonium (bromide salt)
DLS: Dynamic light scattering
ELISA: Enzyme-linked immunosorbent assay
cryo-TEM: Cryogenic transmission electron microscopy
FDA: Food and Drug Administration
H7: Recombinant Influenza A/Shanghai/1/13 (H7N9) Virus Hemagglutinin antigen
HHS: United States Department of Health and Human Services
HRP: Horseradish peroxidase
IACUC: Institutional Animal Use and Care Committee
IFN-α: Interferon α
IFN-γ: Interferon γ
IL: Interleukin
IM: Intramascular
IRB: Institutional Review Board
MSD: MesoScale Discovery
NIH: National Institutes of Health
OD: Optical density
hPBMCs: human peripheral blood mononuclear cells
PDI: Polydispersity index
PVDF: Polyvinylidene fluoride
PAMP: Pathogen-associated molecular pattern
PRR: Pattern recognition receptors
pDCs: Plasmacytoid dendritic cells
ROS: Reactive oxygen species
RP-HPLC: Reverse-phase high performance liquid chromatography
RPMI: Roswell Park Memorial Institute
ssRNA: single stranded RNA
TLR: Toll-like receptor
TNF-α: Tumor necrosis factor α
WFI: sterile water for irrigation
UM: University of Montana
UV/Vis: Ultraviolet/visible spectrum
